# Characterization of the mechanism of action of lanraplenib, a novel spleen tyrosine kinase inhibitor, in models of lupus nephritis

**DOI:** 10.1186/s41927-021-00178-3

**Published:** 2021-03-30

**Authors:** Christopher W. Pohlmeyer, Ching Shang, Pei Han, Zhi-Hua Cui, Randall M. Jones, Astrid S. Clarke, Bernard P. Murray, David A. Lopez, David W. Newstrom, M. David Inzunza, Franziska G. Matzkies, Kevin S. Currie, Julie A. Di Paolo

**Affiliations:** 1 Department of Biology, Gilead Sciences, Inc., 333 Lakeside Dr, Foster City, CA 94404 USA; 2 Department of Drug Metabolism, Gilead Sciences, Inc., Foster City, CA USA; 3 Department of Nonclinical Safety and Pathobiology, Gilead Sciences, Inc., Foster City, CA USA; 4 Department of Clinical Research, Gilead Sciences, Inc., Foster City, CA USA; 5 Department of Chemistry, Gilead Sciences, Inc., Foster City, CA USA

**Keywords:** Spleen tyrosine kinase, SYK inhibition, B-cell receptor, B-cell signaling, Immunohistochemistry, Systemic lupus erythematosus, Lupus nephritis, Inflammation

## Abstract

**Background:**

B cells are critical mediators of systemic lupus erythematosus (SLE) and lupus nephritis (LN), and antinuclear antibodies can be found in the serum of approximately 98% of patients with SLE. Spleen tyrosine kinase (SYK) is a nonreceptor tyrosine kinase that mediates signaling from immunoreceptors, including the B cell receptor. Active, phosphorylated SYK has been observed in tissues from patients with SLE or cutaneous lupus erythematosus, and its inhibition is hypothesized to ameliorate disease pathogenesis. We sought to evaluate the efficacy and characterize the mechanism of action of lanraplenib, a selective oral SYK inhibitor, in the New Zealand black/white (NZB/W) murine model of SLE and LN.

**Methods:**

Lanraplenib was evaluated for inhibition of primary human B cell functions in vitro. Furthermore, the effect of SYK inhibition on ameliorating LN-like disease in vivo was determined by treating NZB/W mice with lanraplenib, cyclophosphamide, or a vehicle control. Glomerulopathy and immunoglobulin G (IgG) deposition were quantified in kidneys. The concentration of proinflammatory cytokines was measured in serum. Splenocytes were analyzed by flow cytometry for B cell maturation and T cell memory maturation, and the presence of T follicular helper and dendritic cells.

**Results:**

In human B cells in vitro, lanraplenib inhibited B cell activating factor-mediated survival as well as activation, maturation, and immunoglobulin M production. Treatment of NZB/W mice with lanraplenib improved overall survival, prevented the development of proteinuria, and reduced blood urea nitrogen concentrations. Kidney morphology was significantly preserved by treatment with lanraplenib as measured by glomerular diameter, protein cast severity, interstitial inflammation, vasculitis, and frequency of glomerular crescents; treatment with lanraplenib reduced glomerular IgG deposition. Mice treated with lanraplenib had reduced concentrations of serum proinflammatory cytokines. Lanraplenib blocked disease-driven B cell maturation and T cell memory maturation in the spleen.

**Conclusions:**

Lanraplenib blocked the progression of LN-like disease in NZB/W mice. Human in vitro and murine in vivo data suggest that lanraplenib may be efficacious in preventing disease progression in patients with LN at least in part by inhibiting B cell maturation. These data provide additional rationale for the use of lanraplenib in the treatment of SLE and LN.

**Supplementary Information:**

The online version contains supplementary material available at 10.1186/s41927-021-00178-3.

## Background

Systemic lupus erythematosus (SLE) is a chronic heterogeneous autoimmune disease, driven by an autoimmune attack on any of several organs, most commonly the skin or kidney. The pathogenesis of SLE is complex, and many pathways involving various immune cell types are dysregulated. Current pharmacotherapy is tailored toward reducing disease activity and preventing end-organ manifestations through broadly immunosuppressive mechanisms. Commonly used treatments include corticosteroids, azathioprine, cyclophosphamide, and mycophenolate, though these treatments may have significant side effects that can limit their use. A more targeted inhibitory profile may reduce off-target effects and improve treatment tolerance.

Evidence from several studies supports a central role of B cells in disease pathology operating through several proposed mechanisms, including cytokine production, antigen presentation to autoreactive T cells, and autoantibody generation and production [1–3]. Considerable effort has been spent developing therapeutics to treat SLE, many of which are designed to modulate B cell prevalence or activity [3–5]. Currently, the only targeted therapy approved for the treatment of SLE is belimumab, a human monoclonal antibody with specificity for B-cell activating factor (BAFF); this therapeutic is not approved for severe manifestations such as lupus nephritis (LN), which occurs in 40–80% of patients with SLE [6], and has a poor overall response rate [7]. The need for improved treatment of SLE and LN has driven interest to explore new targets for therapeutic development. In LN, pathogenesis is driven by deposition of immune complexes (ICs) in glomeruli; the complexes are poorly cleared, leading to acute tissue damage and recruitment of immune cells, which propagate additional damage [8].

Spleen tyrosine kinase (SYK) is an integral component of signaling through multiple immunoreceptor tyrosine-based activation motif (ITAM)-containing immunoreceptors across several different immune cell types, including the B cell receptor (BCR), Fc receptors (FcR), and β2 integrin. Inhibition or deletion of SYK has significant detrimental immune effects [9]. Through BCR signaling, SYK has been demonstrated to play a role in the development [10–12], activation [11, 13, 14], and maturation of B cells [15], as well as antibody production and class switching [15]. SYK has also been demonstrated to be involved in BAFF-mediated B cell survival [16], though its specific role has not been resolved [17]. Additionally, SYK has been shown to be involved in both toll-like receptor (TLR)7- and TLR9-signaling pathways, which can be triggered upon uptake of ICs [18, 19]. Given the role of SYK in both BCR and TLR signaling, it has been proposed that inhibition of SYK could prevent the maturation of autoreactive B cells [20].

SYK dysregulation has been observed in SLE patients. Patients with LN have an infiltration of SYK-expressing cells in the glomeruli [21], and peripheral B cells of SLE patients with active disease exhibit increased levels of SYK phosphorylation [22]. Upon BCR cross-linking, Chang et al. demonstrated an increase in SYK phosphorylation in B cells of SLE patients compared to healthy controls [23]. Additionally, SYK transcript levels are significantly elevated in keratinocytes of patients with cutaneous lupus erythematosus (CLE) compared to healthy control subjects [24].

Several intracellular SYK inhibitors have been developed and evaluated in preclinical lupus models, but none have been evaluated in human clinical trials for SLE or LN [25]. Fostamatinib (R788) successfully ameliorated lupus-like disease in the New Zealand black/white (NZB/W) [26] and Murphy Roths Large/lymphoproliferation (MRL/*lpr*) [27] preclinical mouse models of SLE and LN, although the inhibitor is nonselective and it is unclear if the activity can be solely attributed to inhibition of SYK activity [28]. Fostamatinib and MK-8457 were tested clinically in autoimmune indications and displayed dose-limiting toxicities that limit their development [29–31]; fostamatinib has been approved for immune thrombocytopenia, though it requires monitoring and dose adjustments due to toxicities [32]. Additionally, GSK2646264 is currently being evaluated for topical use in the treatment of CLE (NCT02927457).

Lanraplenib is a novel, selective, oral, once-daily intracellular SYK inhibitor [33]. In the current study, we examined the effects of lanraplenib on primary human B cell functionality and LN-like disease progression in the NZB/W murine model. We characterized the potential mechanism of action of lanraplenib by analyzing renal function, renal histology, systemic cytokine levels, and splenic immune cell populations.

## Methods

### Human B cell in vitro assays

Frozen human peripheral blood mononuclear cells (PBMCs) from healthy donors or individuals with SLE (AllCells) were treated with lanraplenib or dimethyl sulfoxide (DMSO) before stimulation. To measure B cell survival, B cells were treated with human BAFF (R&D Systems, Inc., Minneapolis MN) or RP10; viability was measured using RealTime-Glo™ reagent (Promega Corporation, Madison WI). To measure B cell activation, B cells were stimulated with 20 μg/mL antihuman F (ab’)_2_ immunoglobulin M (IgM) (SouthernBiotech, Birmingham, AL) for 16 h; activation was measured by expression of CD69 and CD86 (BD Biosciences, San Jose, CA) measured by fluorescence-activated cell sorting (FACS) (FACSCanto™ II, BD Biosciences).

Naïve B cells were isolated from freshly collected PBMCs (AllCells) by negative selection (STEMCELL Technologies). They were then treated with lanraplenib or DMSO before stimulation with a stimulation cocktail of F (ab’)_2_ antihuman immunoglobulin D (IgD) (SouthernBiotech), IL-2 and IL-10 (BioLegend®, San Diego, CA), and human MEGACD40L (Enzo Life Sciences, Inc., Farmingdale, NY) for 7 days. Cells were stained for expression of CD3, CD19, and CD27 (BioLegend) before analysis on an LSRFortessa™ X-20 (BD Biosciences). Supernatant antibody concentration was measured using a human antibody isotyping 7-plex immunoassay kit (Invitrogen ProcartaPlex, Carlsbad, CA) read on a FLEXMAP 3D® (Luminex Corporation, Austin, TX).

### NZB/W study: ethical statement

This basic study design and animal usage was approved by Bolder BioPATH’s Institutional Animal Care and Use Committee (IACUC) for compliance with regulations prior to study initiation (IACUC Protocol #BBP-005).

### NZB/W study: study design

Mice were housed at Bolder BioPATH until enrollment in the study at 28 weeks. Mice were assigned to one of 4 groups of 15 mice each (1 positive, 1 negative control group, 2 experimental groups) by distributing mice by body weight and proteinuria equivalently across groups. All mice were bled by retro-orbital sinus, at ages 28, 34, and 40 weeks for plasma for anti–dsDNA antibody analysis (Alpha Diagnostic) and at 29, 31, and 35 weeks for PK plasma. Urine was collected from all mice weekly to measure proteinuria by dipstick measurement. Body weight was measured weekly. Food consumption was measured daily. An overview of the study design is shown in Fig. 2.

### NZB/W study: experimental procedures

Animals were housed 3–5/cage in filtered, pie-shaped polycarbonate cages (Animal Care Systems, Inc.) with wood chip bedding and suspended food and water bottles. Animal care including room, cage, and equipment sanitation conformed to the guidelines cited in the Guide for the Care and Use of Laboratory Animals (Guide, 2011) and the applicable standard operating procedures of Bolder BioPATH. During the acclimation and study periods, animals were housed in a laboratory environment with temperatures ranging 67–76 °F and relative humidity of 30–70%. Automatic timers provided 12 h of light and 12 h of dark. Animals were allowed access ad libitum to Harlan Teklad Rodent Chow and fresh municipal tap water. Mice were dosed orally with lanraplenib (0.075% or 0.25%) or vehicle-administered ad libitum in chow. Positive controls were treated with cyclophosphamide (5 mg/kg) administered daily intraperitoneally.

### NZB/W study: experimental animals

Female NZBWF1/J mice were purchased from The Jackson Laboratory (Bar Harbor, ME) at 20 weeks of age; additional female mice (*n* = 15) that were 14 weeks old on arrival at study week 37 were obtained and used as predisease FACS analysis controls. Mice weighed from 30 to 55 g from arrival at Bolder BioPATH until study initiation. At the end of study, mice were anesthetized with Isoflurane (VetOne) and bled by cardiac puncture for terminal plasma, serum, and whole blood. Spleens and kidneys were also harvested and weighed immediately. Spleens were processed and immunophenotyped by flow cytometry. Kidneys were fixed in 10% neutral buffered formalin before being embedded into paraffin. Kidney sections were stained with hematoxylin and eosin for histologic analysis. For mice that died or were euthanized prior to study termination, terminal clinical scores were carried through to study termination for analysis purpose.

### Serum cytokine concentration

Serum cytokine concentrations were measured with a murine cytokine and chemokine 36-plex immunoassay (Invitrogen ProcartaPlex). Samples were analyzed using a FLEXMAP 3D (Luminex Corporation). Measurements of concentrations of proinflammatory cytokines were analyzed.

### Splenocyte preparation and staining for flow cytometry

Cells were stained for FcR blocking antibodies (BioLegend) before staining with Bcl6, CD3, CD4, CD8, CD11b, CD11c, CD19, CD23, CD44, CD45R/B220, CD62L, CD93, CD138, CD172a, CXCR5, IgM, MHCII (BioLegend), and IgD (Invitrogen) in 4 separate panels. Stained samples were analyzed on a FACSCelesta, an LSR II, or an LSRFortessa X-20 (BD Biosciences). Data were analyzed using FlowJo software.

### RNA extraction and gene expression analysis

Total RNA was extracted from formalin fixed paraffin-embedded scrolls of mouse kidney (Acepix Bioscience, Inc., Hayward, CA). The expression of 93 target genes and 3 housekeeping genes (RPL19, RPL0, and ACTB) were analyzed with standard Taqman assays using Fluidigm Biomark RT-qPCR (bioanalytic and single-cell core at UT Health at San Antonio). Cycle threshold (Ct) data were collected by GE 96 × 96 Standard v2 software. Ct values for each transcript were normalized using the geometric mean of ACTB, RPL19, and RPLP0 Ct values. *P* values were calculated by Mann-Whitney U test. Nominal *P* values are presented.

### Kidney H&E histopathology measurements

Twenty glomeruli per sample were measured to determine mean glomerular diameter and crescent score. Scoring was performed according to methodology previously established [34]. Specifically: Glomerular diameter was scored as follows: 0: > 65 μm; 0.5: 60–70 μm; 1: 71–80 μm; 2: 81–90 μm; 3: 91–100 μm; 4: 101–110 μm; 5: > 110 μm. Crescent percentage was scored as follows: 0: no crescents; 0.5: single glomerulus with crescent; 1: 2–4% of glomeruli with crescents; 2: 5–10% of glomeruli with crescents; 3: 11–25% of glomeruli with crescents; 4: 25–50% of glomeruli with crescents; 5: > 50% of glomeruli with crescents. Protein cast severity was scored as followed: 0: normal tubules; 0.5: affects 1–2% of cortex; 1: affects 3–10% of cortex; 2: affects 11–25% of cortex; 3: affects 26–50% of cortex; 4: affects 51–75% of cortex; 5: affects > 75% of cortex. Interstitial inflammation was scored as follows: 0: no inflammation; 0.5: small focal area of mononuclear cells in pelvis only; 1: occasional small focal accumulation of mononuclear cells affecting > 10% of total interstitium; 2: multifocal mononuclear infiltrates affecting 10–25% of cortex area; 3: multifocal mononuclear infiltrates affecting 26–50% of cortex area; 4: multifocal mononuclear infiltrates affecting 51–75% of cortex area; 5: diffuse mononuclear infiltration affecting 75–100% of cortex area. Vasclitis was scored as follows: 0: no vasculitis; 0.5: one vessel with minimal perivascular infiltrate; 1: 1–2 foci of perivascular infiltrates; 2: 3–4 foci of perivascular infiltrate without necrosis; 3: 5–6 foci of perivascular infiltrate with or without necrosis of vessel wall; 4: 7–8 foci of perivascular infiltrate with or without necrosis of vessel wall; 5: > 8 foci of perivascular infiltrate with extensive necrosis. Summed scores represent sum of each of 5 individually measured criteria above.

### IgG staining and scoring

Kidney sections were stained with Alexa Fluor 647 conjugated anti-mouse IgG antibody (Cell Signaling Technology®, 4410S) and DAPI. IgG deposition in glomeruli was scored into 4 grades based on the amount of fluorescence signal observed in each glomerulus: Grade 1: < 25% deposit; Grade 2: 25–49% deposit; Grade 3: 50–74% deposit; Grade 4: ≥75% deposit. Approximately 200 glomeruli were scored from each mouse.

### Statistical analysis

Statistics were calculated in Prism (GraphPad Software Inc.) unless otherwise specified. Pairwise comparisons were made by testing for normal distribution of data. Data normality was tested by D’Agnostine-Pearson omnibus normality test. Normally distributed data were analyzed by ANOVA and non-normally distributed data were analyzed by a Kruskal-Wallis test. ANOVA was corrected for multiple comparisons by the Holm-Sidak method; Kruskal-Wallis was corrected with a Dunn’s multiple comparison test. For analyses with samples below the limit of detection, the limit of detection was used as the quantified value. Effective concentration (EC)_50_ values were calculated using a 4-parameter variable slope nonlinear regression fitted by least squares. Nonlinear best fit regressions were calculated using GraphPad Prism.

## Results

### SYK inhibition blocks B cell functions in vitro

**Fig. 1:**
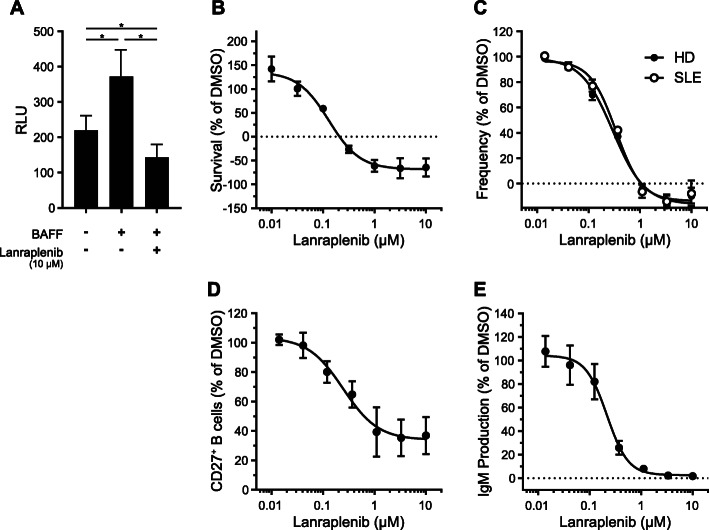
Lanraplenib inhibits human B-cell survival, activation, maturation, and antibody production of in vitro. **a-b** Lanraplenib-treated B cells from healthy donors (HD) were stimulated with recombinant B-cell activating factor (BAFF) (250 ng/mL) for 48 h. Survival was measured by RealTime Glo. Comparison of unstimulated and BAFF-stimulated B cells with and without lanraplenib treatment are shown in **a**. Survival of untreated cells without BAFF stimulation was subtracted from all wells to generate dose response curve in **b**. EC_50_: 130 nM; *n* = 6. **c** Lanraplenib-treated B cells isolated from blood of HD or individuals with SLE were stimulated with F (ab’)_2_ anti-IgM antibody (20 μg/mL) for 16 h. Activation was measured by the expression of CD69. EC_50_: 298 nM (HD), 340 nM (SLE); *n* = 3 donors per group. **d** Lanraplenib-treated naïve B cells (CD27^−^) from HD were stimulated with a cocktail of interleukin (IL)-2 (50 U/mL), IL-10 (10 ng/mL), CD40L (100 ng/mL), and F (ab’)_2_ anti-IgD antibody (1 μg/mL) for 7 days. Maturation was measured by the expression of CD27. EC_50_: 245 nM; *n* = 4. **e** Antibody production was measured by the concentration of IgM in supernatant samples from **c**. EC_50_: 216 nM; *n* = 4. Error bars represent standard error of the mean (SEM). * Indicates *P* < 0.05 as calculated by repeated measures one-way ANOVA

The effect of SYK inhibition on primary human B cells was characterized in vitro. In culture, human B cells die rapidly; however, when stimulated with recombinant BAFF, their survival is prolonged (Fig. 1a). Lanraplenib treatment of B cells prior to BAFF stimulation dose-dependently reduced B cell survival with an EC_50_ value of 130 nM (Fig. 1a and b). Interestingly, lanraplenib inhibited BAFF-mediated B cell survival to a level below the unstimulated control (Fig. 1a), demonstrating that lanraplenib can inhibit basal survival signaling in human B cells [35]. Lanraplenib also inhibited BAFF-mediated survival of mouse BALB/c splenic B cells in a dose-dependent fashion (see Supplemental Fig. 1A, Additional File).

As SYK is a critical component of the BCR signaling cascade, the effect of lanraplenib on BCR-mediated B cell activation was measured. Primary human B cells isolated from the blood of healthy donors or individuals with SLE were treated with lanraplenib, and the expression of CD69 was measured following BCR engagement. We observed a dose-dependent inhibition of CD69 expression with EC_50_ values of 298 nM and 340 nM, respectively (Fig. 1c).

To test the effect of lanraplenib on B cell maturation in vitro, lanraplenib-treated CD27-naïve human B cells were stimulated with a cocktail of interleukin (IL) 2, IL10, and CD40L, and a BCR-stimulating polyclonal antibody (F (ab’)_2_ anti-IgD). B cell maturation was blocked by SYK inhibition as measured by expression of CD27 with an EC_50_ value of 245 nM (Fig. 1d). Additionally, lanraplenib inhibited the secretion of IgM in a dose-dependent fashion with an EC_50_ value of 216 nM (Fig. 1e). Similar results were observed when murine splenic B cells were stimulated with a cocktail of IL4, CD40L, and BCR-stimulating monoclonal antibody (see Supplemental Figs. 1B and C, Additional File).

### Lanraplenib ameliorates LN-like disease in NZB/W mice

**Fig. 2:**
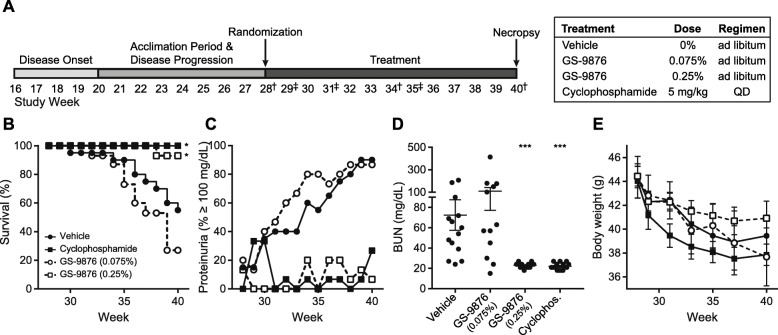
Lupus-like phenotype in NZB/W mice is ameliorated by lanraplenib treatment. **a** Experimental study design to test the efficacy of lanraplenib. Lanraplenib was formulated in chow at the indicated percentages. Proteinuria was measured every week starting at week 28. **b** Kaplan-Meier curve showing the percentage of surviving mice at each time point; *n* = 15 mice per group at week 28. **c** Percentage of mice with proteinuria (≥ 100 mg/dL) at each time point. Treatment groups are stylized as in **b**. **d** Blood urea nitrogen measurement at week 40. Each point represents an individual mouse at the time of sacrifice. Mean ± SEM are indicated for each treatment group. Statistics were calculated comparing the vehicle control group to each other group using a Kruskal-Wallis test. **e** Mean body weight of mice in each group ± SEM. Treatment groups are stylized as in **b**. Food consumption was measured daily; no differences in food consumption were observed across any treatment groups. * Indicates *P* < 0.05. *** indicates *P* < 0.0005. ^†^Indicates blood draws for dsDNA Ab measurement. ^‡^Indicates blood draws for PK measurement

To evaluate the efficacy of lanraplenib in an in vivo system, lanraplenib was evaluated in the NZB/W murine preclinical model of SLE. These mice recapitulated several clinical elements of SLE [36]. The study scheme for evaluating lanraplenib in NZB/W mice is depicted in Fig. 2a. Mice dosed at 0.075 and 0.25% in chow had calculated average SYK target coverage (AUC) of approximately 50 and 80%, respectively as measured by a pervanadate PD assay; due to the nature of continuous food consumption and subsequent continuous dosing, target coverage remained consistent over the 24-h period measured (data not shown). The lanraplenib (0.075%)-treated group did not show a statistically significant difference in survival compared to the vehicle control group at week 40 (Fig. 2b). Groups treated with either lanraplenib (0.25%) or cyclophosphamide (5 mg/kg/day) showed a statistically significant improvement in overall survival at week 40 compared to the vehicle-treated group (*P* = 0.044 and 0.010, respectively).

NZB/W mice develop significant glomerulonephritis and proteinuria. While approximately 90% of mice in the vehicle-treated group developed proteinuria (≥100 mg/dL) by week 40, lanraplenib (0.25%) treatment prevented the development of proteinuria to a similar extent as cyclophosphamide (5 mg/kg/day) treatment (Fig. 2c). Due to the inherent limitations associated with measuring proteinuria by dipstick measurements, we also measured blood urea nitrogen to characterize kidney activity. Both lanraplenib (0.25%) and cyclophosphamide treatment (5 mg/kg/day) significantly reduced blood urea nitrogen levels, another indicator of improved kidney function (Fig. 2d). Treatment with lanraplenib (0.25%) or cyclophosphamide reduced kidney weight, spleen weight, and blood cholesterol levels, suggesting the general improvement of lupus disease phenotypes (see Supplemental Figs. 2A, B, and C, Additional File). While treatment with either lanraplenib (0.25%) or cyclophosphamide improved overall survival and kidney function, mice treated with cyclophosphamide lost more body weight compared to the vehicle-treated mice, whereas mice treated with lanraplenib (0.25%) lost less weight than vehicle-treated mice (Fig. 2e), suggesting that lanraplenib was better tolerated by these mice.

### Kidney morphology is improved by SYK inhibition

**Fig. 3:**
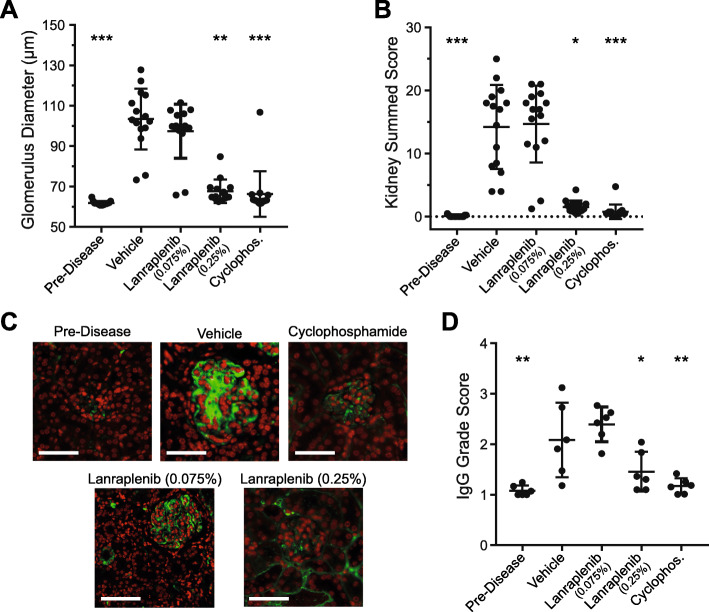
Kidney morphology is improved by lanraplenib treatment in NZB/W mice. **a** Average glomerulus diameter of kidney sections. Each point represents an individual mouse at the time of sacrifice. **b** Summed histology score of kidney sections. **c** Representative glomerular IgG staining for each group. Green: anti-IgG staining; red: DAPI. Scale bar represents 100 μm. **d** Summary of glomerular IgG staining. Scoring criteria are specified in Fig. S3. Mean ± SD are indicated for each treatment group. Statistics were calculated comparing the vehicle control group to each other group using either an ANOVA or Kruskal-Wallis test. *Indicates *P* < 0.05. ** Indicates *P* < 0.005. *** Indicates *P* < 0.0005

Renal histology was evaluated to better characterize the effect of lanraplenib treatment. Because NZB/W mice spontaneously develop LN-like disease, young, prediseased mice were used as controls. Glomerular diameter, protein cast severity, interstitial inflammation, vasculitis, and frequency of glomerular crescents were measured in sections of renal tissue harvested from mice from each treatment group (see Supplemental Fig. 3). Vehicle-treated mice had significantly increased glomerular diameter compared to predisease mice (Fig. 3a). Treatment with lanraplenib (0.075%) had minimal effect on glomerular diameter. Treatment with lanraplenib (0.25%) or cyclophosphamide significantly reduced glomerular diameter. The summed scores for the kidney morphologic features measured, shown in Fig. 3b, are significantly increased in vehicle-treated mice as compared to the predisease mice. The lanraplenib (0.075%) treatment group showed no improvement in kidney score, whereas the kidney scores were significantly reduced in both the lanraplenib (0.25%) and cyclophosphamide treatment groups toward predisease levels.

As antibody deposition in the kidney can drive nephritis and glomerular destruction, IgG deposition in glomeruli across treatment groups was quantified by grading the degree of IgG deposition into 4 categories (grade 1–4) (see Supplemental Fig. 3, Additional File). Representative glomerular IgG staining in each treatment is shown in Fig. 3c. We observed an increase in IgG deposition in glomeruli based on the presence of disease (vehicle-treated group vs predisease group) (Fig. 3d). Lanraplenib (0.075%) treatment did not reduce glomerular IgG deposition, whereas lanraplenib (0.25%) or cyclophosphamide treatment significantly reduced glomerular IgG deposition as measured by IgG grade score (Fig. 3d).

### Lanraplenib normalizes transcription of genes in kidney sections

To further characterize the effect of lanraplenib on the kidney, renal transcripts of select genes that encode cytokines and proteins involved in apoptosis, apoptotic cell clearance, and structural integrity were quantified. Several of the transcripts were significantly more prevalent in renal tissue harvested from vehicle-treated mice compared to those of predisease mice (see Supplemental Fig. 4A, Additional File) and were significantly less prevalent in the renal tissue harvested from either the lanraplenib (0.25%)- or cyclophosphamide-treated mice compared to those of the vehicle-treated mice. A similar magnitude of change in the prevalence of transcripts was observed when the vehicle-treated group was compared to each of the predisease, lanraplenib (0.25%)-treated, and cyclophosphamide-treated groups (see Supplemental Fig. 4B, Additional File).

### SYK inhibition reduces serum proinflammatory cytokine concentrations

**Fig. 4:**
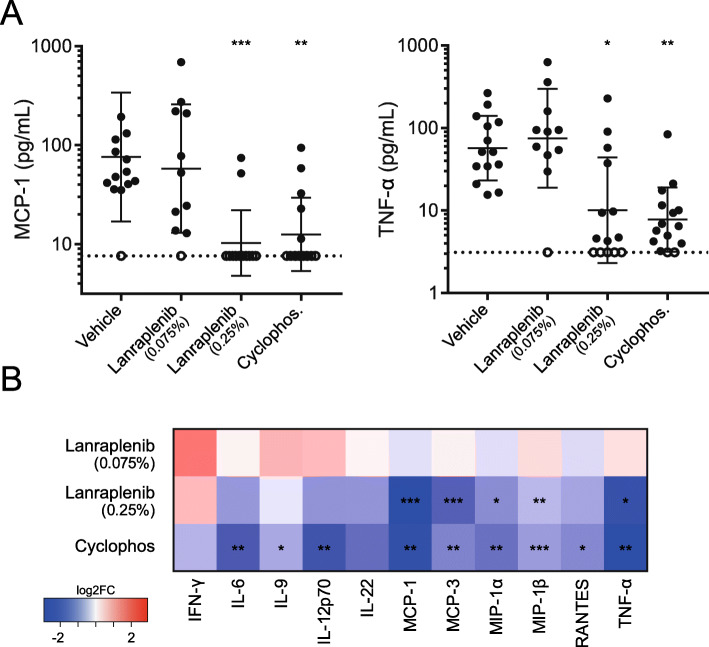
Serum proinflammatory cytokine concentrations are reduced in lanraplenib-treated NZB/W mice. **a** Geometric mean (± geo standard deviation) serum concentration of MCP-1 (left) and TNF-α (right) of individual mice. Each point represents an individual mouse. Limit of detection of assay is represented by a dashed line. Open circles indicate serum concentrations below the limit of detection. Statistics were calculated comparing the vehicle control group to each other group using Kruskal-Wallis tests. **b** Heat map summarizing the geometric mean fold-change comparing the vehicle control group to each indicated treatment group. *Indicates *P* < 0.05. **Indicates *P* < 0.005. ***Indicates *P* < 0.0005

To gain an understanding of the overall phenotype of disease amelioration with lanraplenib treatment, the concentration of 18 proinflammatory cytokines in the serum of NZB/W mice was measured at age 40 weeks. Of the 18 cytokines measured, the concentrations of 7 were below the limit of detection (see Supplemental Fig. 5, Additional File). Treatment with lanraplenib (0.075%) had no effect on serum concentrations of monocyte chemotactic protein (MCP)-1 or tumor necrosis factor (TNF)-α (Fig. 4a); however, the concentration of both cytokines was significantly lower in the lanraplenib (0.25%) and cyclophosphamide treatment groups. Treatment with either lanraplenib (0.25%) or cyclophosphamide showed an overall trend toward a reduction in the proinflammatory cytokine concentrations. A statistically significant reduction was observed in 5 serum cytokines with lanraplenib (0.25%) and 9 with cyclophosphamide, suggesting that lanraplenib has a more targeted inhibitory profile while cyclophosphamide potentially has a broader immunosuppressive effect. A heat map comparing the fold-change of the geometric mean of cytokine concentration between the vehicle-treated group to each of the lanraplenib (0.075%)-, lanraplenib (0.25%)-, and cyclophosphamide-treated groups is shown in Fig. 4b.

### Lanraplenib inhibits maturation of splenic B cells in NZB/W mice

**Fig. 5:**
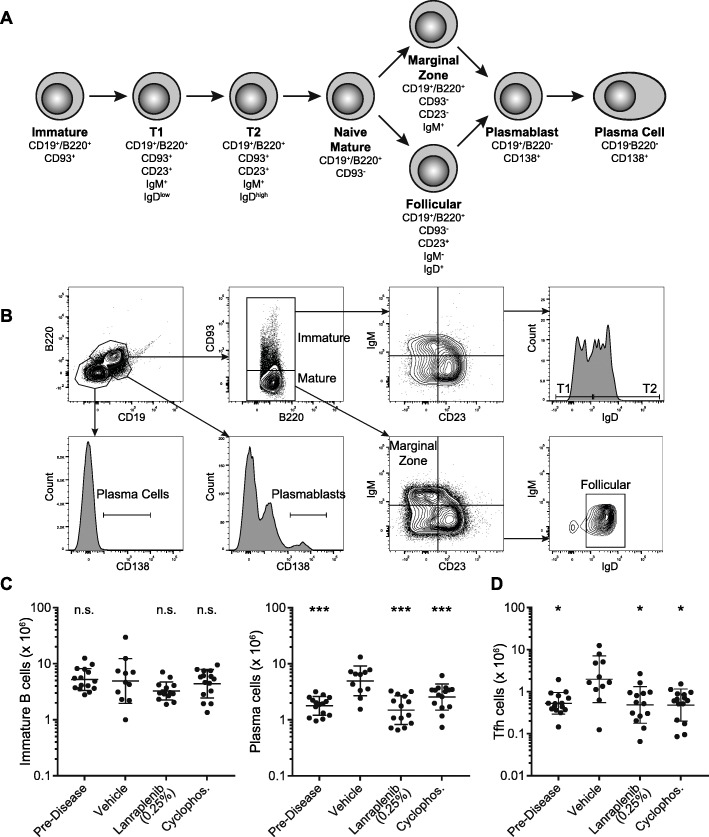
B cell maturation is prevented by lanraplenib in the spleens of NZB/W mice. **a** Schematic of B cell maturation in the spleen. Surface markers used in this study to define each phase of B cell maturation are shown. **b** Representative flow cytometry gating scheme. **c** Geometric mean (± geo standard deviation) of the abundance of immature B cells (left), plasma cells (middle), and Tfh cells (right) within the spleens of mice at the end of the study. Cell counts were normalized to spleen mass. Each point represents an individual mouse. Statistics were calculated comparing the vehicle control group to each other group using either an ANOVA or Kruskal-Wallis test. *Indicates *P* < 0.05. ***Indicates *P* < 0.0005

To determine if SYK inhibition could inhibit B cell maturation in vivo, the presence of B cells in different stages of maturation in the spleens of NZB/W mice was quantified. Figure 5a shows a simplified schematic of the stages of B cell maturation that were measured and the cell surface markers used to identify each population by flow cytometry. Figure 5b shows the gating scheme used to identify B cells in the different stages of maturation. There was no detectable change in the abundance of immature B cells due to disease progression; however, vehicle-treated mice had a significantly increased count of plasma cells compared to predisease mice (Fig. 5c). Treatment with lanraplenib (0.25%) or cyclophosphamide did not have an effect on immature B-cell count but significantly reduced the development of plasma cells. Supplemental Figs. 6A and B, Additional File, show the effect of disease and treatment with lanraplenib (0.25%) or cyclophosphamide within each stage of B cell maturation shown in Fig. 5a. Overall, lanraplenib treatment modulated the abundance of B cells in later stages of maturation without affecting the prevalence of B cells in earlier stages of maturation.

Additionally, T follicular helper (Tfh) cells were found to be more abundant in vehicle-treated mice compared to predisease mice (Fig. 5c). Treatment with lanraplenib (0.25%) or cyclophosphamide reduced the number of Tfh cells in the spleen. The disease was also observed to affect the ratio of naïve to memory CD4 and CD8 T cells, as well as the prevalence of CD11b^+^ dendritic cells within the spleen; treatment with lanraplenib (0.25%) or cyclophosphamide had restorative effects on all of these populations (see Supplemental Fig. 7, Additional File) back to predisease levels.

## Discussion

In this study, we sought to evaluate the efficacy and characterize the mechanism of action of lanraplenib, a selective oral SYK inhibitor, in human B cells and in vivo in the NZB/W murine model of SLE. Our results demonstrate that lanraplenib-mediated inhibition of SYK can block in vitro B cell activation and maturation of isolated primary human B cells, which are believed to be a driver of disease. Additionally, lanraplenib blocked the progression of LN-like disease in NZB/W mice. Altogether, these data suggest that the effect of lanraplenib in preventing disease progression was at least in part due to inhibition of B cell maturation.

B cells are critical mediators of SLE pathology and have generated research interest. B cells have multiple functions that affect SLE pathogenesis, including cytokine production, antigen presentation to autoreactive T cells, and autoantibody generation and production [1–3]. Antinuclear antibody (ANA) production is a hallmark of SLE; the most commonly found ANA recognizes dsDNA and can be found in the serum of 30–70% of patients with SLE [4, 37, 38]. ANAs can form ICs with nuclear materials released during apoptosis [39, 40], necrosis [39], and NETosis [41]. In healthy individuals, ICs are bound by complement proteins and are quickly cleared [42]; however, many SLE patients have defects in one or more complement pathway components [43], leading to reduced clearance of ICs and resultant type III hypersensitivity [44].

In the present study, we did not observe a change in serum dsDNA antibody concentrations between 28 and 40 weeks of age with disease progression. It is likely that the effects of lanraplenib or cyclophosphamide on serum dsDNA antibody concentration were negligible due to the initiation of treatment after dsDNA antibody production had already plateaued. Bahjat et al. observed a similar lack of dsDNA antibody change in vehicle-treated NZB/W after 28 weeks of age [26]. We have previously tested the effects of a molecule in the same class as lanraplenib in the MRL/lpr murine model of lupus. Between 14 and 20 weeks of age, vehicle-treated MRL/lpr mice showed significantly increased serum dsDNA antibody levels; however, mice treated with either a SYK inhibitor or cyclophosphamide had significantly lower serum dsDNA antibody concentration compared to the vehicle-treated mice at week 20 (see Supplemental Fig. 8, Additional File). These data suggest that SYK inhibition can prevent the development of autoantibodies in vivo. Additionally, by reducing B cell activation and maturation, inhibition of SYK may prevent the intra- and interantigenic epitope spreading observed in SLE patients [45–48].

BCR signaling is necessary for B cell activation and maturation into antibody-producing cells [49], and SYK is a central player in BCR signal transduction. Upon binding to doubly phosphorylated ITAM of the BCR [50, 51], SYK is auto- [52] and transphosphorylated [53, 54], and recruits signaling molecules including BLNK [55, 56], PI3K [57, 58], PLC-γ [59, 60], and VAV [61, 62]. Inhibition of SYK phosphorylation results in dissociation of SYK from the ITAM and internalization of the BCR [63]. Conditional SYK knockout mice have defects in B cell development [10, 12] and produce decreased levels of antigen-specific antibodies after exposure to antigen [15]. In this study, we observed an inhibition in maturation of B cells within the spleens of NZB/W mice treated with lanraplenib (0.25%).

SYK has a role in many biological pathways that could contribute to SLE pathogenesis, and its modulation may have a therapeutic benefit beyond affecting B cell function and maturation. Through its dual Src homology 2 domains, SYK has been demonstrated to interact with ITAMs downstream of integrins [64, 65], FcR [66], and other signaling molecules [67–69]. SYK is expressed in many different cell types that have ITAM-containing signaling receptors, including neutrophils, conventional dendritic cells (DCs), plasmacytoid DCs (pDCs), and others.

In neutrophils, SYK signaling occurs after β2 integrin activation during rolling adhesion [70, 71] and transcellular migration [72]. In addition, proinflammatory cytokines and chemokines, including MCP1, MCP2, and macrophage inflammatory protein (MIP)-1α, are involved in neutrophil recruitment; B cells and myeloid cells can produce MCP1, MCP2, and MIP-1α via TLR7 and TLR9 signaling pathways after endocytosis or phagocytosis of nucleic acid-containing ICs through FcR molecules [73–78]. Furthermore, stimulation of pDCs with serum isolated from SLE patients can induce production of several proinflammatory cytokines, including TNF-α, through the uptake of nucleic acid-containing ICs via FcR molecules [79, 80]. SYK inhibition has been demonstrated to block FcR-dependent IC uptake by classical DCs [81]. In the present study, treatment with lanraplenib reduced the serum concentration of MCP1, MIP-1α, and TNF-α in NZB/W mice (Fig. 4b).

The T cell receptor (TCR) signals through several ITAMs [82, 83], and it has been proposed that SYK plays a role in TCR signaling in SLE patients [84–87]. However, the role of SYK in TCR signaling in NZB/W mice is unknown. The observed effects on splenic T cells with lanraplenib treatment (decreased T cell memory maturation and reduced Tfh abundance) are likely secondary effects and not attributable to direct SYK inhibition of T lymphocyte signaling. We hypothesize that the inhibition of proinflammatory cytokine production observed in this study reduced CD11b^+^ DC activation and subsequent activation of T cells and differentiation into Tfh [88, 89]. This could further support the role of SYK inhibition in treatment of Sjögren’s syndrome.

SYK has a central role in diverse functional activities across multiple cell types, including BCR signal transduction, neutrophil adhesion, and FcR-mediated endocytosis. In this study, we observed a reduction in splenic B cell maturation with lanraplenib treatment.

## Conclusion

These results demonstrate that lanraplenib has a protective role against the development of LN-like disease in NZB/W mice. SYK inhibition may have multiple protective roles in SLE and LN, including but not limited to inhibition of B cell maturation. Characterization of B cells in various stages of maturation may be beneficial to monitor in clinical studies of lanraplenib.

## Supplementary Information

**Additional file 1: Supplemental Figure1.** Lanraplenib inhibits murine B cell survival, maturation, and antibody production in vitro. (A) Lanraplenib-treated splenic CD43^−^ B cells were stimulated with recombinant BAFF (1 ng/mL) for 72 h. Survival was measured by RealTime Glo. Survival measurement of untreated cells without BAFF stimulation was subtracted from all wells. EC50: 121 nM. *n* = 3. (B) Lanraplenib-treated splenic CD43- B cells were stimulated with a cocktail of IL4 (10 U/mL), CD40L (200 ng/mL), and F (ab’)2 anti-IgD antibody (1 μg/mL) for 7 days. Maturation was measured by the expression of CD27. EC50: 169 nM. *n* = 3. (C) Antibody production was measured by the concentration of IgM in supernatant samples from C. EC50: 462 nM. *n* = 3. Error bars represent SD. **Supplemental Figure2.** Improved phenotype of NZB/W F1 mice with lanraplenib treatment. Kidney weight (A), spleen weight (B), and blood cholesterol levels (C) of each group at time of sacrifice. Each point represents an individual mouse. Mean ± SD are indicated for each treatment group. Statistics were calculated comparing the vehicle control group to each other group using either ANOVA or Kruskal-Wallis tests. *P < 0.05, **P < 0.005, ***P < 0.0005. **Supplemental Figure 3.** H&E images and glomerular IgG deposition scoring. (A) Representative H&E images for each treatment group. G: glomeruli; V: vasculitis; large arrow: protein casts in tubules. (B) Glomerular IgG staining was scored by eye read and binned into 4 scores. Grade 1: < 25% deposit; Grade 2: 25–49% deposit; Grade 3: 50–74% deposit; Grade 4: ≥75% deposit. Green: anti-IgG staining; red: DAPI. **Supplemental Figure 4.** Transcriptional changes in renal samples of NZB/W F1 mice are restored to prediseased levels after lanraplenib treatment. (A) Volcano plot showing fold-change and nominal *P* values of transcripts of apoptotic proteins, proteins involved in apoptotic cell clearance, cytokines, and structural proteins. Each point represents an individual gene transcript. Horizontal line represents a 10^− 4^ *P* value cutoff. Vertical lines represent a 2-fold change cutoff. (B) Heatmap summarizing fold-change comparing each group to the vehicle control group. Transcripts quantified are specified below plot. **Supplemental Figure 5.** Changes in serum proinflammatory cytokine concentrations in lanraplenib-treated NZB/W F1 mice. Geometric mean (± Geo SD) serum concentration of all cytokines that were above (A) or below the limit of detection (B). Each point represents and individual mouse. Statistics were calculated comparing the vehicle control group to each other group using Kruskal-Wallis tests. **Supplemental Figure 6.** Splenic B cells have a less-matured phenotype in lanraplenib-treated NZB/W F1 mice. Geometric mean (± Geo SD) of abundance of B cells in each step of the maturation process within the spleens of mice at the end of study. Cell counts were normalized to spleen mass. Each point represents an individual mouse. Statistics were calculated comparing the vehicle control group to each other group using either ANOVA or Kruskal-Wallis tests. *P < 0.05, **P < 0.005, ***P < 0.0005. **Supplemental Figure 7.** Reduced abundance of T and dendritic cells in the spleens of lanraplenib-treated NZB/W F1 mice. Mean ratio of naïve:memory CD4^+^ (left) and CD8^+^ (middle) at the end of study. Geometric mean (± Geo SD) of abundance of CD11b^+^ DC (right) within the spleens of mice at the end of study. Cell counts were normalized to spleen mass. Each point represents an individual mouse. Statistics were calculated comparing the vehicle control group to each other group using an ANOVA test. *P < 0.05, **P < 0.005, ***P < 0.0005. **Supplemental Figure 8.** SYK inhibition blocks dsDNA antibody development in MRL/*lpr* mice. Average dsDNA antibody titers in each treatment group (*n* = 15 animals per group). Treatment was initiated at age 14 weeks . *P < 0.05, ***P < 0.0005.

## Data Availability

The datasets used and/or analysed during the current study are available from the corresponding author on reasonable request.

## Abbreviations

ANA: Anti-nuclear antibody
BAFF: B cell activating factor
BCR: B cell receptor
CLE: Cutaneous lupus erythematosus
Ct: Cycle threshold
DC: Dendritic cell
DMSO: Dimethyl sulfoxide
dsDNA: Double-stranded DNA
EC: Effective concentration
FACS: Fluorescence-activated cell sorting
FcR: Fragment, crystallizable receptor
IC: Immune complexes
IgD: Immunoglobulin D
IgG: Immunoglobulin G
IgM: Immunoglobulin M
IL: Interleukin
ITAM: Immunoreceptor tyrosine-based activation motif
LN: Lupus nephritis
MCP: Monocyte chemotactic protein
MIP: Macrophage inflammatory protein
MRL/lpr: Murphy Roths Large/lymphoproliferation
NZB/W: New Zealand black/white
PBMCs: Peripheral blood mononuclear cells
PD: Pharmacodynamic
pDC: Plasmacytoid dendritic cells
SEM: Standard error of the mean
SLE: Systemic lupus erythematosus
SYK: Spleen tyrosine kinase
TCR: T cell receptor
Tfh: T follicular helper
TLR: Toll-like receptor
TNF: Tumor necrosis factor
