# Supplementary material for: Characterization of the mechanism of action of lanraplenib, a novel spleen tyrosine kinase inhibitor, in models of lupus nephritis
Source: BMC Rheumatol. 2021 Mar 30;5:15. doi: 10.1186/s41927-021-00178-3 (PMC8008554; doi:10.1186/s41927-021-00178-3)

Title: Supplementary data to manuscript titled “Characterization of the Mechanism of Action of Lanraplenib, a Novel Spleen Tyrosine Kinase Inhibitor, in Models of Lupus Nephritis”

**Supplemental Figure 1.** **Lanraplenib inhibits murine B cell survival, maturation, and antibody production in vitro. (A)** Lanraplenib-treated splenic CD43^-^ B cells were stimulated with recombinant BAFF (1 ng/mL) for 72 hours. Survival was measured by RealTime Glo. Survival measurement of untreated cells without BAFF stimulation was subtracted from all wells. EC50: 121 nM. n = 3. **(B)** Lanraplenib-treated splenic CD43- B cells were stimulated with a cocktail of IL4 (10 U/mL), CD40L (200 ng/mL), and F (ab’)2 anti-IgD antibody (1 μg/mL) for 7 days. Maturation was measured by the expression of CD27. EC50: 169 nM. n = 3. **(C)** Antibody production was measured by the concentration of IgM in supernatant samples from C. EC50: 462 nM. n = 3. Error bars represent SD.

**Supplemental Figure 2. Improved phenotype of NZB/W F1 mice with lanraplenib treatment**. Kidney weight **(A)**, spleen weight **(B)**, and blood cholesterol levels **(C)** of each group at time of sacrifice. Each point represents an individual mouse. Mean ± SD are indicated for each treatment group. Statistics were calculated comparing the vehicle control group to each other group using either ANOVA or Kruskal-Wallis tests. **P*<0.05, ***P* <0.005, ****P*< 0.0005.

**Supplemental Figure 3. H&E images and glomerular IgG deposition scoring. (A)** Representative H&E images for each treatment group. G: glomeruli; V: vasculitis; large arrow: protein casts in tubules. **(B)** Glomerular IgG staining was scored by eye read and binned into 4 scores. Grade 1: <25% deposit; Grade 2: 25%–49% deposit; Grade 3: 50%–74% deposit; Grade 4: ≥75% deposit. Green: anti-IgG staining; red: DAPI.

**Supplemental Figure 4. Transcriptional changes in renal samples of NZB/W F1 mice are restored to prediseased levels after lanraplenib treatment. (A)** Volcano plot showing fold-change and nominal *P* values of transcripts of apoptotic proteins, proteins involved in apoptotic cell clearance, cytokines, and structural proteins. Each point represents an individual gene transcript. Horizontal line represents a 10^–4^ *P* value cutoff. Vertical lines represent a 2-fold change cutoff. **(B)** Heatmap summarizing fold-change comparing each group to the vehicle control group. Transcripts quantified are specified below plot.

**Supplemental Figure 5. Changes in serum proinflammatory cytokine concentrations in lanraplenib-treated NZB/W F1 mice.** Geometric mean (± Geo SD) serum concentration of all cytokines that were above **(A)** or below the limit of detection **(B)**. Each point represents and individual mouse. Statistics were calculated comparing the vehicle control group to each other group using Kruskal-Wallis tests

**Supplemental Figure 6. Splenic B cells have a less-matured phenotype in lanraplenib-treated NZB/W F1 mice.** Geometric mean (± Geo SD) of abundance of B cells in each step of the maturation process within the spleens of mice at the end of study. Cell counts were normalized to spleen mass. Each point represents an individual mouse. Statistics were calculated comparing the vehicle control group to each other group using either ANOVA or Kruskal-Wallis tests. **P*< 0.05, ***P*< 0.005, ****P*< 0.0005.

**Supplemental Figure 7. Reduced abundance of T and dendritic cells in the spleens of lanraplenib-treated NZB/W F1 mice.** Mean ratio of naïve:memory CD4^+^ (left) and CD8^+^ (middle) at the end of study. Geometric mean (± Geo SD) of abundance of CD11b^+^ DC (right) within the spleens of mice at the end of study. Cell counts were normalized to spleen mass. Each point represents an individual mouse. Statistics were calculated comparing the vehicle control group to each other group using an ANOVA test. **P*<0.05, ***P*<0.005, ****P*<0.0005.

**Supplemental Figure 8. SYK inhibition blocks dsDNA antibody development in MRL/*lpr* mice.** Average dsDNA antibody titers in each treatment group (n=15 animals per group). Treatment was initiated at age 14 weeks . **P*<0.05, ****P*<0.0005.

**Supplemental Figure 1**


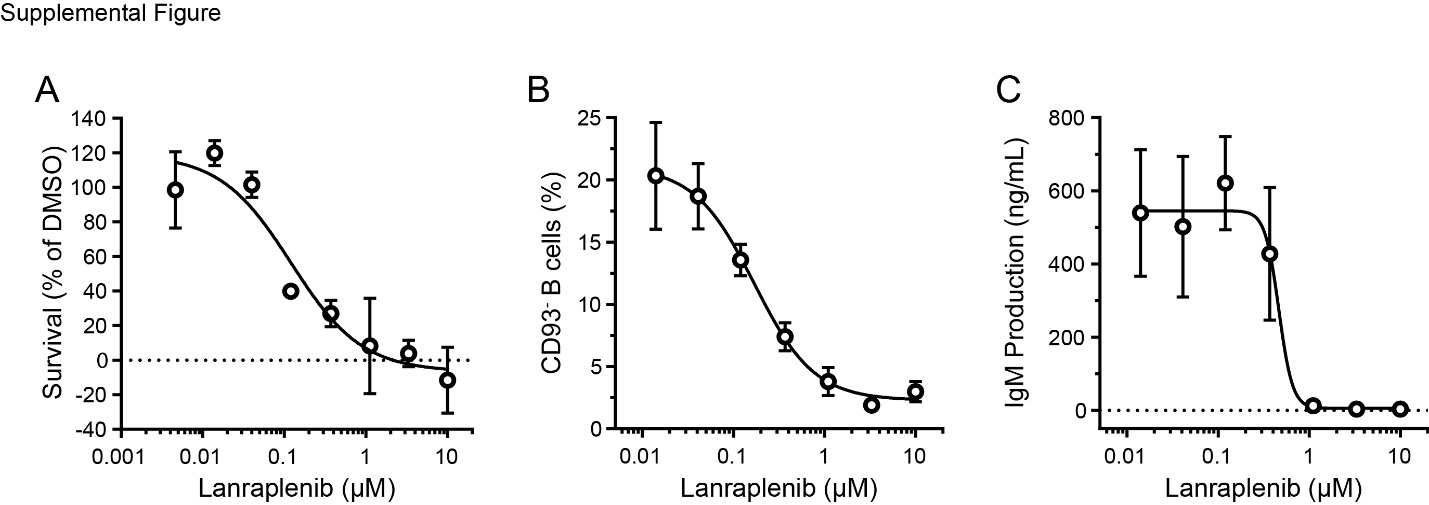


**Supplemental Figure 2**


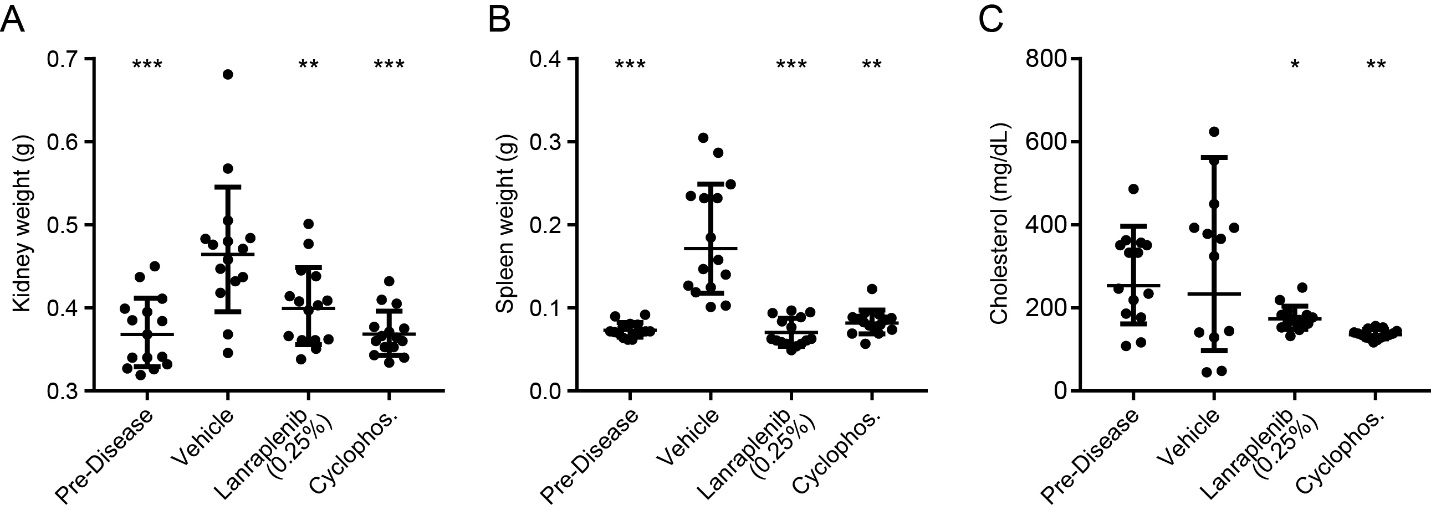


**Supplemental Figure 3**


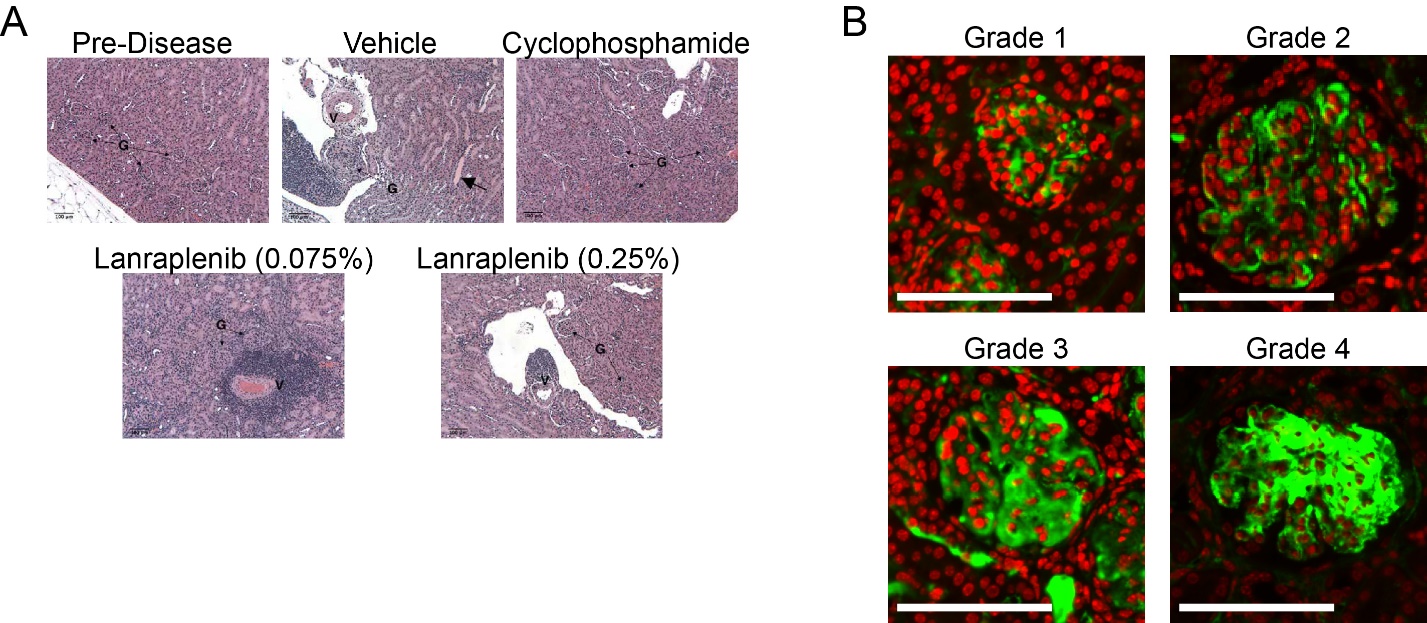


**Supplemental Figure 4**


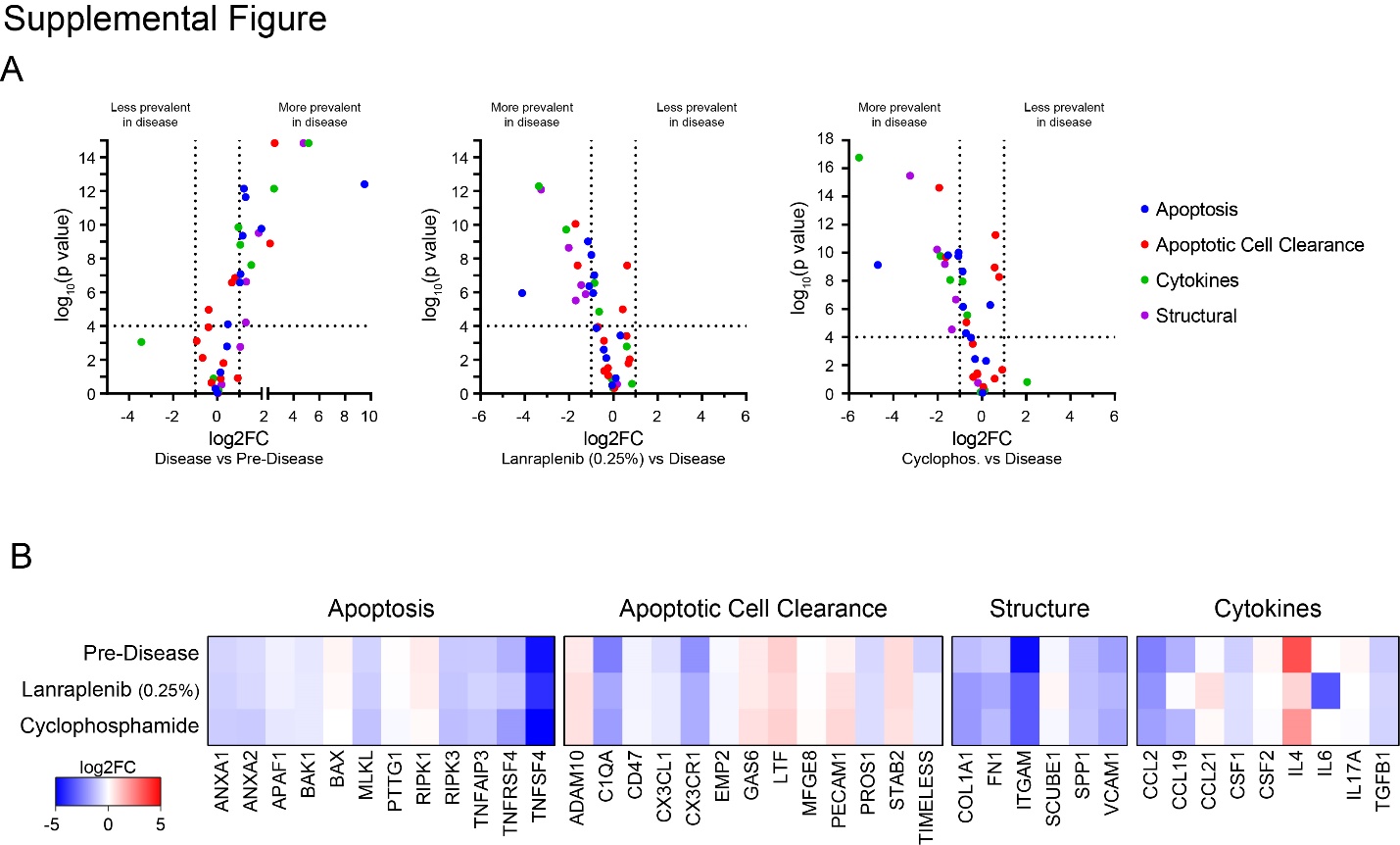


**Supplemental Figure 5**


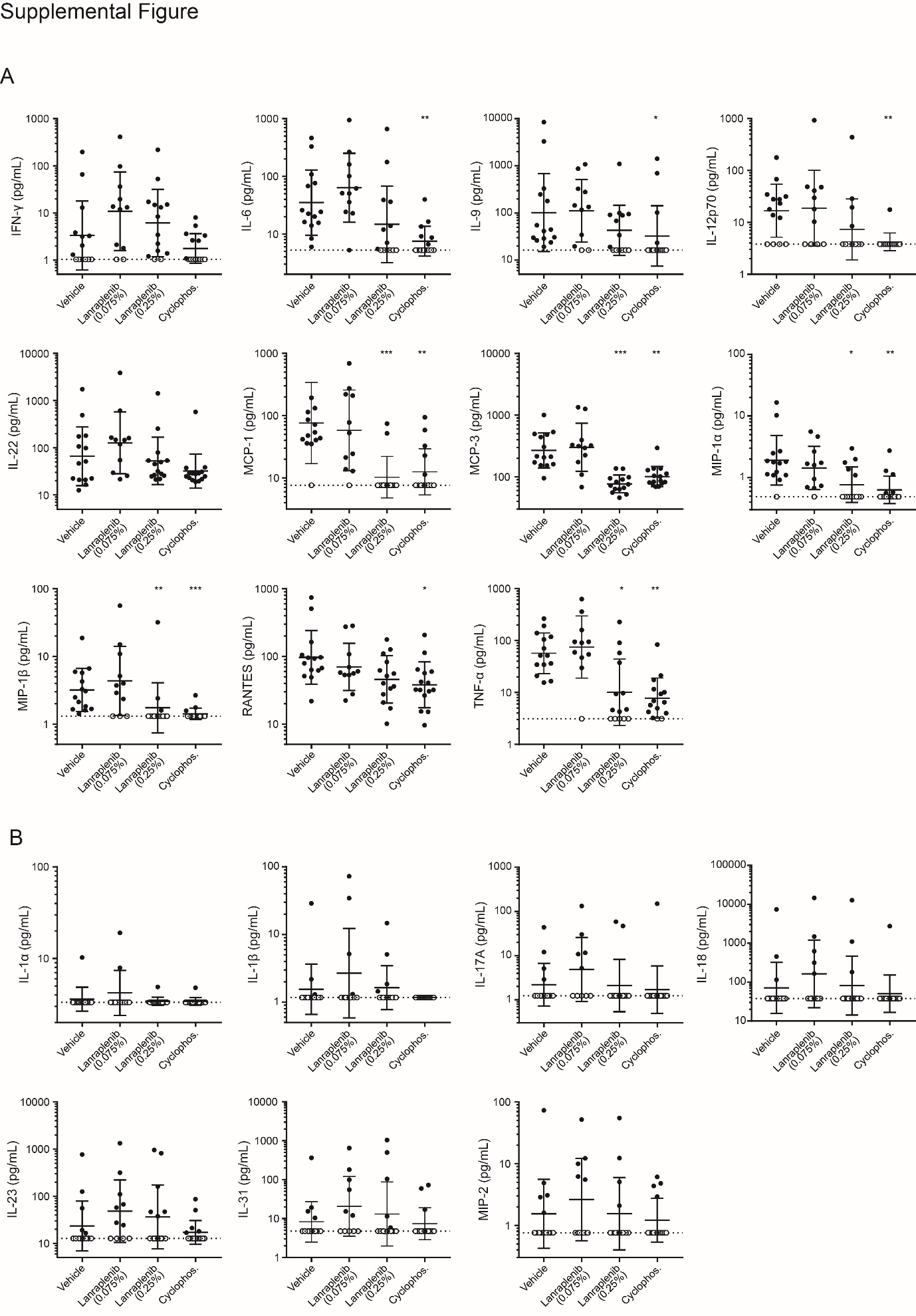


**Supplemental Figure 6**


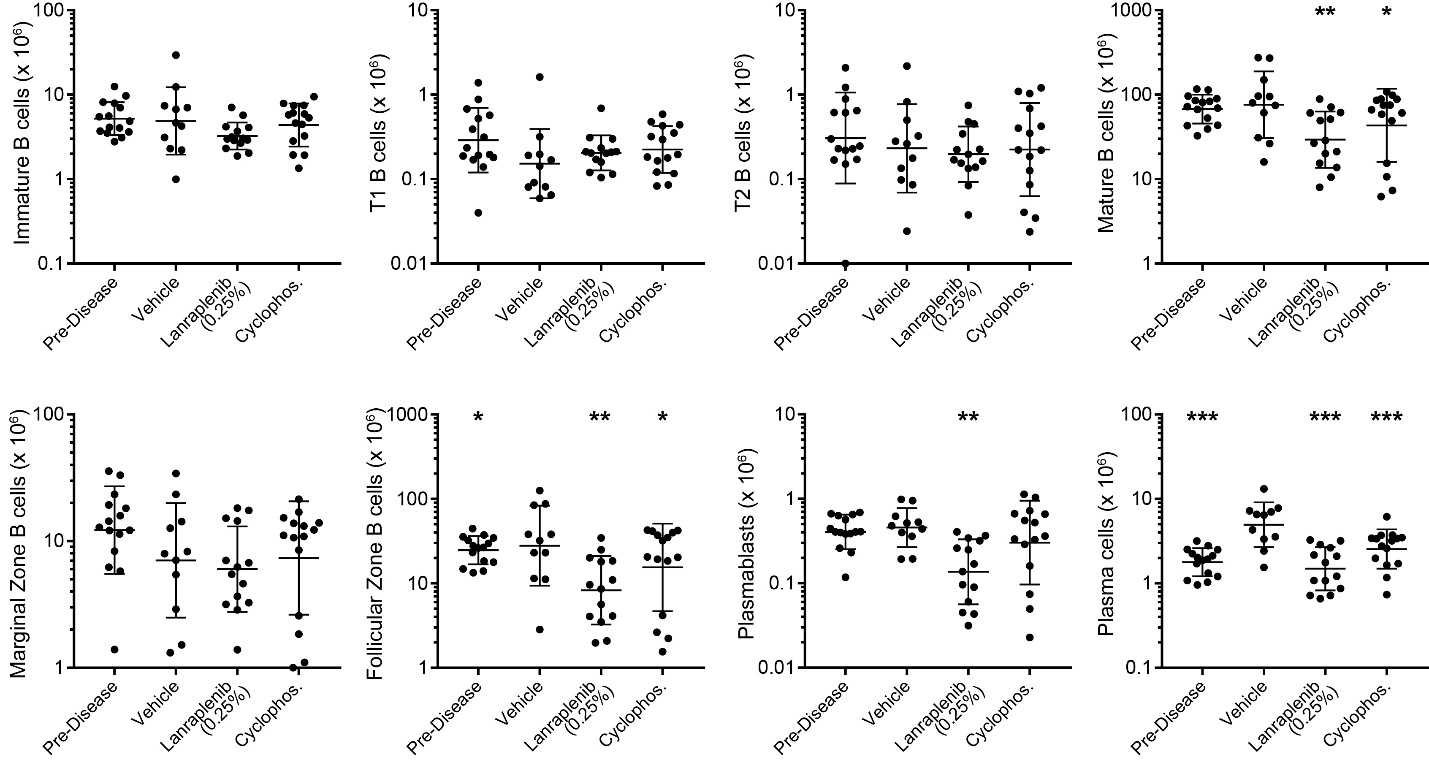


**Supplemental Figure 7**


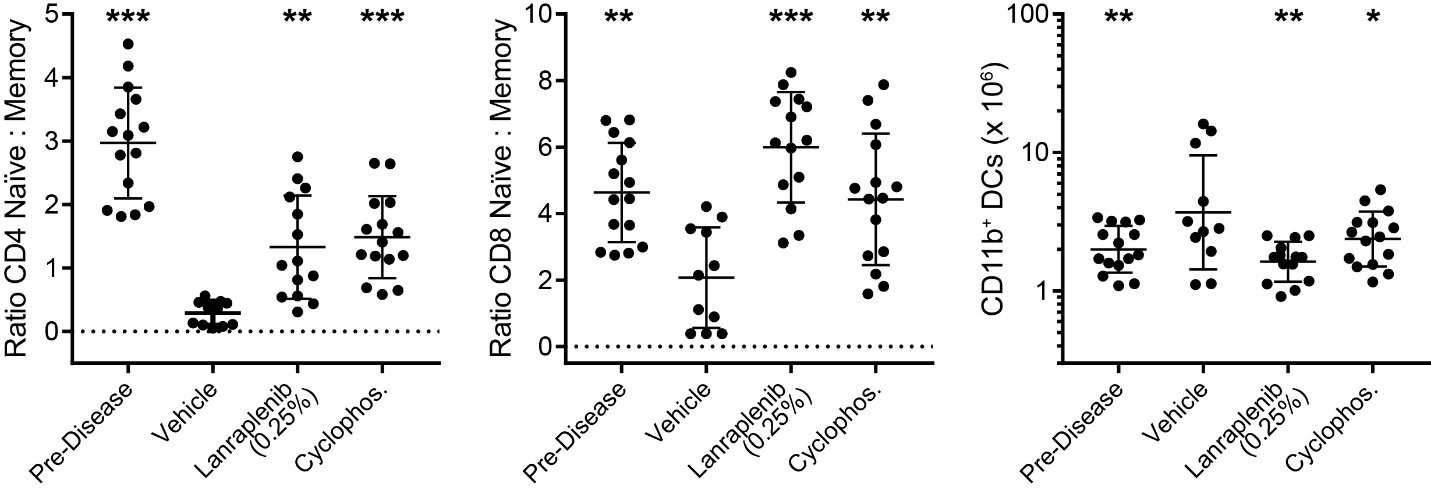


**Supplemental Figure 8**


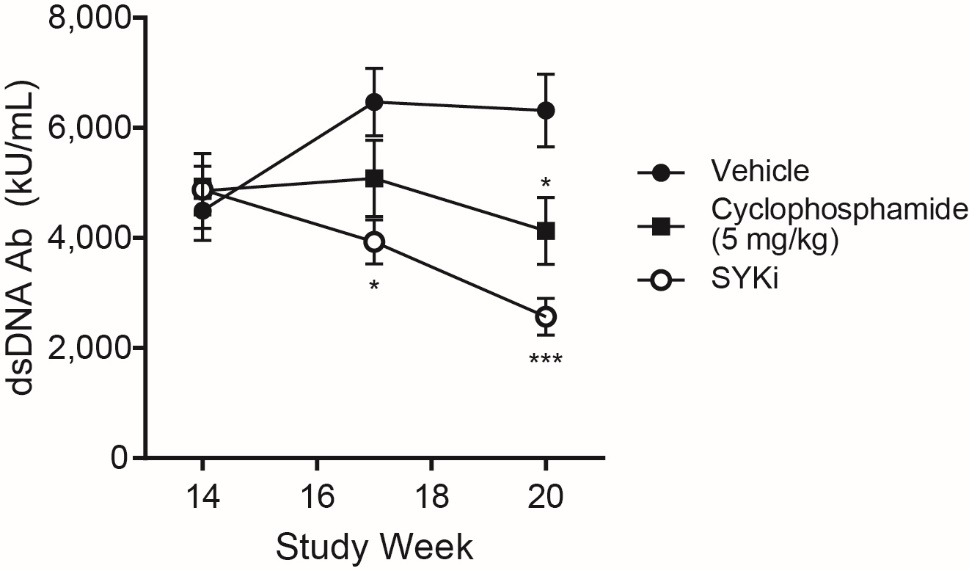

Supplement: Supplementary file 1 — Additional file 1: Supplemental Figure1. Lanraplenib inhibits murine B cell survival, maturation, and antibody production in vitro. (A) Lanraplenib-treated splenic CD43− B cells were stimulated with recombinant BAFF (1 ng/mL) for 72 h. Survival was measured by RealTime Glo. Survival measurement of untreated cells without BAFF stimulation was subtracted from all wells. EC50: 121 nM. n = 3. (B) Lanraplenib-treated splenic CD43- B cells were stimulated with a cocktail of IL4 (10 U/mL), CD40L (200 ng/mL), and F (ab’)2 anti-IgD antibody (1 μg/mL) for 7 days. Maturation was measured by the expression of CD27. EC50: 169 nM. n = 3. (C) Antibody production was measured by the concentration of IgM in supernatant samples from C. EC50: 462 nM. n = 3. Error bars represent SD. Supplemental Figure2. Improved phenotype of NZB/W F1 mice with lanraplenib treatment. Kidney weight (A), spleen weight (B), and blood cholesterol levels (C) of each group at time of sacrifice. Each point represents an individual mouse. Mean ± SD are indicated for each treatment group. Statistics were calculated comparing the vehicle control group to each other group using either ANOVA or Kruskal-Wallis tests. *P < 0.05, **P < 0.005, ***P < 0.0005. Supplemental Figure 3. H&E images and glomerular IgG deposition scoring. (A) Representative H&E images for each treatment group. G: glomeruli; V: vasculitis; large arrow: protein casts in tubules. (B) Glomerular IgG staining was scored by eye read and binned into 4 scores. Grade 1: < 25% deposit; Grade 2: 25–49% deposit; Grade 3: 50–74% deposit; Grade 4: ≥75% deposit. Green: anti-IgG staining; red: DAPI. Supplemental Figure 4. Transcriptional changes in renal samples of NZB/W F1 mice are restored to prediseased levels after lanraplenib treatment. (A) Volcano plot showing fold-change and nominal P values of transcripts of apoptotic proteins, proteins involved in apoptotic cell clearance, cytokines, and structural proteins. Each point represents an individual gene t [file 41927_2021_178_MOESM1_ESM.docx]
